# Prisoners regulating prisons: Voice, action, participation and riot

**DOI:** 10.1177/17488958221101997

**Published:** 2022-06-16

**Authors:** Gillian Buck, Philippa Tomczak

**Affiliations:** University of Chester, UK; University of Nottingham, UK

**Keywords:** Participation, prison regulation, prisoner voice, riots, Strangeways, voluntary sector

## Abstract

Prisoners are a critical source of prison regulation around the world, but regulation *by* (rather than *of*) prisoners remains little analysed. In this article, we utilise the 1990 riots at HMP Strangeways (England), as a case study of prisoners (re)shaping imprisonment. We examine prisoners’ roles in these riots and subsequent cross-sectoral regulatory activities. We innovatively use the four-phase process of translation from actor-network theory to guide document analysis of (1) Lord Woolf’s official inquiry into the riots and (2) the voluntary organisation Prison Reform Trust’s follow-up report. We explore how participatory approaches could inform prison regulation through (former) prisoners partnering with external regulators throughout the processes of identifying problems and solutions to establish broader alliances seeking social change.

## Introduction

Prison regulation seeks to steer the flow of events and behaviour to improve institutional performance and hold key personnel responsible (Braithwaite et al., 2007). Regulation encompasses sanctioning and supporting activities including education, persuasion, litigation, and prizes, which can influence conditions and treatment in institutions (Tomczak, 2021). Regulation can safeguard rights and has transformed some public services (Smith, 2009), enhancing wellbeing through quality standards in, for example, health, food and housing (Braithwaite, 2017: 25). A narrow view of prison regulation encompasses only ‘official’ external monitoring and inspection mechanisms (van Zyl Smit, 2010), and directing others in this way can imply a powerful external authority interfering in a top-down fashion (Braithwaite, 2017). Drahos and Krygier (2017: 4) advocate a broader view of regulation, seeing the state as part of a regulatory network in which tasks are distributed among multisectoral actors. A broad view of prison regulation encompasses actors including voluntary organisations and (bereaved) families (Tomczak, 2021), along with staff and prisoners within institutions who are potentially influential from the ‘bottom up’ (Darke, 2018; Levit, 2008; Norman, 2021).

In this article, we examine how prisoner actions, in combination with the activities of external regulatory or oversight bodies, have shaped imprisonment. In turn, we begin to highlight the untapped potential of participatory prison regulation, advocating a broad view of prison regulation which explicitly includes prisoners themselves. Using the actor-network theory (ANT) four-phase process of translation (Tomczak, 2016), we map how the 1990 prison riots in England were translated into thought and action through the official Woolf and Tumim report (1991) and voluntary sector ‘Strangeways 25 Years On’ report. Our approach innovatively reveals the agency of prisoners in initiating and shaping formal regulatory processes over time and, simultaneously, how prisoner voices and actions were limited. While (former) prisoners have a long history of shaping imprisonment, their actions are often written out of official discourses (Sim, 1994). Paradoxically, prisoners are now increasingly mobilised as ‘service users’ who can inform and improve systems (Buck et al., 2021). Understanding how prisoners have been agents of change *and* marginalised in official discourse could enable more effective future ‘acts in concert’ (Arendt, 1958). Arendt argues that power is the ability not just to act but to act *in concert with others* to create something new (Bay, 2012; Habermas, 1994).

### A narrow view: ‘Official’ prison regulation

England and Wales’ prisons have a complex ‘official’ regulatory framework spanning local Independent Monitoring Boards, the national Inspectorate of Prisons, the Prisons and Probation Ombudsman, and international European and United Nations (UN) oversight committees (van Zyl Smit, 2010). Regulators can form an important ‘counterweight to potential abuse of the special powers of the state’ (Hood et al., 1999: 116). Yet, important questions remain regarding the ‘quality and purpose of official information’ (Hancock and Liebling, 2013: 111), and the production processes involved. A common complaint is that regulators’ recommendations are not implemented (Stark, 2020), leaving prisoners vulnerable to abuse (Dolovich, 2021).

The Woolf and Tumim inquiry (1991) into the 1990 prison disturbances triggered by riots at HMP Strangeways was an official inquiry, convened by a government minister and led by a professional expert. This inquiry is argued to have had ‘the greatest impact on prison conditions in modern times’, prompting a new approach to maintaining order in England’s prisons (van Zyl Smit, 2010: 533–534) which was explicitly focused on transparent decision making and procedural fairness in disciplinary and complaint procedures (Sparks et al., 1996). Innovatively for the time, Lord Woolf invited prisoners to present evidence to the inquiry by writing letters. Woolf’s strategy illustrates an early attempt to facilitate some prisoner participation in the production of official discourse, being ‘very different from the philosophy and methodology behind previous state inquiries where [. . .] the Home Office view prevailed from the beginning’ (Sim, 1994: 35).

### A broader view: Participatory regulation

International research illustrates that prisoners actively shape institutions. Prisoners are sometimes the only source of governance in Latin American prisons, developing alternative rules and forms of negotiated order where official governance is insufficient to meet their needs (Darke, 2018; Skarbek, 2016). In Nicaraguan prisons, co-governance is organised between authorities and powerful prisoners, who can both instrumentalise others to control prison life (Weegels, 2020). Organised, ethnically segregated groups in Californian prisons govern prisoner activities and adjudicate conflict (Skarbek, 2016).

Collaborative, participatory approaches can facilitate problem solving through dialogue between diverse parties and by leveraging the unique knowledges and different perspectives of multiple actors (Holley, 2017; Shapiro, 2003). Directly involving key actors in governance may foster stakeholder ownership, giving greater voice to marginalised interests rather than relying on bureaucratic expertise (Holley, 2017). Recent European policies have encouraged greater citizen participation in the coproduction of public services (Weaver, 2019). Yet, research on prisoners’ democratic participation is scarce (Brosens, 2019) and there is little scholarly exploration of regulation *by* (rather than *of*) prisoners.

This article positions the Strangeways riots and the external regulation that they triggered as pivotal examples of prisoners regulating prisons. We provide a revisionist account, writing prisoners back into prison regulation by highlighting that the riots were prisoner led and examining the participatory elements of Woolf and Tumim (1991) and the Prison Reform Trust (PRT) report. First, we summarise the Woolf and PRT reports that underpin our analysis. Next, we outline the increasing importance of prisoner participation and voice in criminal justice, considering their regulatory potentials and constraints. We then outline our methodology before detailing findings, examining the ways that riot, official inquiry and voluntary sector reporting facilitated and limited prison regulation by prisoners.

## The Woolf Inquiry

On 1 April 1990, prisoners at HMP Strangeways (now HMP Manchester) began the longest and most devastating riot in British penal history (Sim, 1994: 2). By 6 April, the Home Office had appointed Lord Justice Woolf to lead a public inquiry into the events leading up to the disturbances and actions taken to conclude them (Woolf and Tumim, 1991: 28). The ‘Strangeways’ riot lasted 25 days, alongside which

serious riots broke out in five other prisons and various forms of disruption occurred in more than thirty establishments across England and Wales. As a result of the events at Strangeways one prisoner lost his life and 147 prison officers and 47 inmates were injured. (Sim, 1994: 2)

These riots occurred amid the broader context of confrontation in prisons worldwide from the 1960s, which challenged brutalising conditions and alienation in the modern prison (Sim, 1994).

Drawing upon consultations with prisoners, staff, and a range of stakeholders working in and around prisons, Woolf made 12 principal recommendations, including that prison numbers should not exceed certified levels; all inmates have access to sanitation; the prison estate be split into smaller, more manageable units; and standards of justice be improved through independent adjudication and complaint proceedings. These recommendations were well received in Parliament and more broadly (Morgan, 1991). While having dire consequences for prisoners, staff and families, the 1990 prison riots were a relatively accessible means of protest and triggered an official inquiry (Woolf and Tumim, 1991) which highlighted poor conditions and treatment and re-emphasised prisoners’ rights to justice and sanitation (Player and Jenkins, 1994). Material changes included more generous visit allowances, an end to routine censorship of prisoners’ letters, installation of telephones, and an end to slopping out of human waste from prison cells lacking a flush toilet (Morgan, 1992). Significantly, the riots also stimulated a more effective complaints system with external oversight:

Woolf [. . .] recommended the introduction of an independent element in the prisons complaints system, through the appointment of a complaints adjudicator [. . . which] resulted in the establishment in 1994 of the Prisons Ombudsman for England and Wales. (Seneviratne, 2012: 340)

Complaints are now among the most institutionalised and widely available mechanisms for prisoner participation. A fair and effective complaints process is considered integral to stable prisons (Woolf and Tumim, 1991). Participation through complaint can facilitate voice, fairness, legitimacy, dignity, and wellbeing (Weaver, 2019), potentially releasing pent-up frustration, which can avert self-harm, suicide, unrest, violence, and riots (Carl, 2013; Woltz, 2020). Complaining can also initiate systemic improvement (Banwell-Moore and Tomczak, in press). However, problems endure in England and Wales. There is ongoing warehousing of people with complex problems in overcrowded, old, and unsatisfactory buildings (Eady, 2007; Tomczak and Bennett, 2020) and restricted toilet access for those without in-cell sanitation (Day et al., 2015).

## The PRT report

Twenty-five years after the Strangeways riots, PRT published a report highlighting the legacy of the Woolf inquiry. Drawing upon prison inspections, government and voluntary sector reports, PRT reviewed progress made and reversed against each of Woolf’s 12 recommendations (Day et al., 2015). This retrospective report serves as ‘knowledge recall’, recalling a history of lessons (not) learned (Stark, 2020). It also outlines how broader networks of actors (e.g. charities, inspectors, staff collectives, parliamentary committees) can communicate concerns and exert pressure over time.

## Prisoner voice and participation

There is a long, if often-unacknowledged, history of prisoners attempting to regulate prisons, which have resulted in material changes. Strategies have included written critiques, litigation, protest and complaints. Nellis (2002) documents influential (former) prisoner writings, including Joan Henry’s (1952) autobiography *Who Lie in Gaol*, which inspired a film and its national tour, attracting journalists to write about penal reform. Peter Wildeblood’s (1955) *Against the Law* added momentum to the campaign to decriminalise homosexuality. Trevor Hercules’ (1989) *Labelled Black Villain* was serialised in The Observer, giving national prominence to a Black perspective on prison and critiquing the prison’s failure to rehabilitate. Leech (1995) wrote a handbook for prisoners and co-founded *Unlock*, a pressure group comprising ex-prisoners which seeks to improve the lives of people with criminal records (Nellis, 2002: 438–441).

Through litigation such as judicial reviews prisoners, acting in concert with legal professionals, can challenge the lawfulness of government decisions. Mark Leech brought dozens of legal challenges while serving and changed the law, for example, through the 1993 judgement that Section 33.3 of the Prison Rules 1964 was unlawful as it did not allow for unscreened legal correspondence (Scott, 2013). In 2014, prisoner Barbara Gordon-Jones brought a case to the High Court, with assistance from the Howard League for Penal Reform, which declared Justice Secretary Grayling’s ban on sending books to prisoners in England and Wales unlawful.^1^ In the United States, litigation can be used to divert disabled people from prisons through the *Americans with Disabilities Act* (Balaban, 2017). However, published writing and legal action require levels of cultural (and often material) capital that are not available to many prisoners.

More broadly, notions of participation have become central to realising more democratic, sustainable public services which better respond to human needs (Bovaird, 2007). Across social and criminal justice services, peoples’ lived experiences are increasingly mobilised in service delivery and strategy development (Buck et al., 2021). Prisoner ‘voice’ is key to such participation and has clear, yet underdeveloped potential to enhance prison regulation. ‘Voice’ is a political concept emanating from the citizen participation paradigm (Healy, 2017). ‘Prisoner voice’ involves prisoners giving an account of their experiences (e.g. through writings), having a say in who governs them (e.g. in-prison and national elections; Brown, 2008) and direct action, such as complaints or litigation (Woltz, 2020). Participation can improve wellbeing (Weaver, 2019) and counter the dehumanisation and stigmatisation of prisoners (Aresti et al., 2016).

The right to actively participate in services is recognised in law and policy. The *Human Rights Act 1998* empowers service users to challenge professional decisions made without their participation. *The Framework for Patient and Public Participation in Health and Justice Commissioning* (NHS England, 2017) and *Transforming Rehabilitation* in criminal justice (MoJ, 2013) both highlighted the importance of participation. *Valuing People* (Department of Health, 2001) promotes the active involvement of people with learning difficulties in decisions that affect them, including service developments locally and nationally, and 32% of prisoners have a learning disability or difficulty (Skills Funding Agency, 2017). Article 50 of the *European Prison Rules* stipulates that ‘subject to the needs of good order, safety and security, prisoners shall be allowed to discuss matters relating to general conditions of imprisonment and shall be encouraged to communicate with the prison authorities about these matters’ (Brosens, 2019: 466).

Nascent examples of participatory prison regulation activities are primarily organised by the voluntary sector, including prison councils and policy networks. In England and Wales, the *User Voice* organisation was founded by former prisoner Mark Johnson, developing ‘councils’ in prisons, probation, and youth offending teams to give opportunities for criminalised people to have their say and offer solutions (User Voice, 2014). Prison councils can provide a space for ‘watching the watchers’ (Schmidt, 2020: 188), potentially exposing abuses of power and enhancing transparency. Outcomes attributed to prison councils include the provision of in-cell phones, calmer environments, and improved visit areas (Weaver, 2019). PRT’s *Prisoner Policy Network* seeks to enable prisoners to influence policy (PRT, 2022) and has produced several free, publicly available reports from consultations with serving prisoners, documenting concerns, and proposing (collective) solutions.

Although prisoners are increasingly called upon to inform improvements to criminal justice, and prisoners have long attempted to regulate prisons, there are significant constraints upon meaningful participation. The risk management and security priorities within prisons do not align with ideologies underpinning participatory developments, including psychoanalytic notions of liberation through speech and political ideals of empowerment (Brich, 2008). Prisoner participation in regulation can meet resistance and obstruction fuelled by distrust and concerns about the erosion of prison officer authority (Weaver, 2019). Prisoner perspectives can be marginalised and subjugated, even within forums seeking inclusive participation. In 1971, Michel Foucault founded the *Groupe d’information sur les prisons* (GIP), aiming ‘to enable prisoners to speak out on prison issues and decide for themselves what should be done about them’ (Brich, 2008: 26). The group was commended for giving prisoners ‘the voice they were denied’ and had some material successes, leading to the creation of the *Comité d’action des prisonniers*/Prisoners’ Action Committee, which fought for prisoners’ rights through the 1970s (Brich, 2008: 27). However, prisoners were constrained by the methodology and agenda of the GIP’s academic founders. Philosophers designed prisoner questionnaires and wrote the commentary on results. This methodology privileged French speaking, literate, articulate prisoners, and encased their experiences within an interpretive framework employed for Foucault’s own radical aims; ‘inevitably . . . channelling, moulding and mediating inmates’ discourse’ (Brich, 2008: 46). More recently, Buck (2020) outlined a similar process of capture within peer-led criminal justice services. Criminalised people entered previously professional spaces, voicing lived experiences and challenging exclusionary practices. However, peer practitioners were only grudgingly accepted, subject to additional scrutiny and governed by established professional standards (Buck, 2020).

Even within ‘participatory’ structures, (former) prisoners occupy a ‘subaltern’ position, from which the capacity to access power is radically obstructed (Morris, 2010: 8). Prisoners’ social construction as othered victimisers rather than victims or ‘one of us’ (Sloan Rainbow, 2018: 264) impedes them being ‘heard’ beyond accounts which fit established notions or ‘titillate the prison voyeur’ (Warr, 2012: 142). Such constructions allow the criminalised other to exist only as a component of the listener’s experience (Buber, 1985). However, Butler (2021) argues more optimistically:

When the precarious expose their living status to those powers that threaten their very lives, they engage in a form of persistence that holds the potential to defeat [. . .] aims [. . .] to cast those on the margins as dispensable. (p. 24)

For (former) prisoners to participate meaningfully in prison regulation, they must have platforms to enter ‘dialogues’, which involve openness to the fullness of the other’s experience (Buber, 1985). Hence, those listening and speaking must be open to diverse perspectives, suspending stereotypes and sensation.

In these introductory sections, we have examined prisoner voice as an increasing feature of criminal justice, as a right in law, and as a feature of diverse regulation efforts, including writings, litigation, protest and complaint. We also considered limitations including the penal context, differing agendas, and the marginalised position from which prisoners speak. We now outline our methodological approach, before presenting findings, including how prisoner participation in regulation has evolved and what official prison regulators could learn from these changing forms of involvement.

## Actor-network theory and case study methodology

Our aim was to understand the varied ways that prisoners participated in prison regulation over time. Using the Strangeways riot as a case study, we undertook document analysis of the Woolf and Tumim inquiry (1991) and PRT report to map how prisoner actions prompted an official inquiry, contributed to formulating recommendations and – to some degree – supported their implementation. Processual social ontology presumes that everything in the social world is continually being made, remade and unmade (Abbott, 2016). The processual focus on emergence contrasts with, for example, well-trodden Foucauldian notions of regimes and governmental technologies, and valuably counters the dystopias which often prevail in criminological scholarship (Zedner, 2002).Our methodological bracing is taken from ANT, a sociological approach examining the processual emergence of agency, knowledge and organisation, which has been little used within criminology. ANT usefully illustrates how governing operates through heterogeneous human and nonhuman actors (e.g. reports, policy documents, cell keys), and that existing forms of power and organisation can always be reconfigured. ANT highlights that each member (e.g. prisoner, prison officer, charity, prison building) of a network (e.g. the criminal justice system) is actively involved in the translation of thought and action, giving rise to struggles, accommodations, alliances, and separations (Tomczak, 2016; Carrabine, 2000). ANT’s process of translation (Callon, 1986) enables scholars to trace how diverse actors translate phenomena into resources and those resources into more powerful actor-networks (Tomczak, 2016).

The four inter-related phases of translation are problematisation, interessement, enrolment and mobilisation. During ‘problematisation’, the project sponsor seeks to define a problem and interest other actors by defining the means of resolution (Tomczak, 2016). ‘Interessement’ involves the sponsor attempting to stabilise the identities of other actors, who can submit to the initial plan, negotiate terms or define their interests differently (Callon, 1986). ‘Enrolment’ sees the agreeable actors accepting the roles and interests defined by the project sponsor. Inscription often occurs during enrolment, with negotiated commitments being inscribed into the shared memory and stabilised through artefacts such as contracts (Tomczak, 2016: 63). Finally, ‘mobilisation’ is the point at which an actor becomes the spokesperson for an actor-network, speaking for other actors (Sage et al., 2011: 286). Spokespersons, such as journalists and Lord Woolf, are powerful macro actors who can translate the interests, roles and relations of the entire actor-network (Callon and Latour, 1981; Tomczak, 2016: 64).

Callon’s (1986) concept of ‘translation’ was used to structure our document analysis. We mapped prisoner participation in the Strangeways riot and subsequent reporting through three translations. The first translation was principally bottom-up and we examined local interactions between protesting prisoner sponsors, the prison and the media. The second, principally top-down translation begun with the commission of the Woolf inquiry, a problematisation by the Home Office in response to prisoner actions. Third, the PRT report translated artefacts produced by Woolf’s inquiry and documents produced in the subsequent 25 years, to problematise progress and act as spokesperson by making recommendations. This method enabled us to trace prisoner participation, processes of definition and (in)action, and the mechanisms by which recommendations entered penal policy and practice over time (or did not). We used the following four questions to frame our analysis: (1) Did prisoner actors identify problems and possible solutions? (2) did prisoner actors submit to the problematisation, refuse to take part, or negotiate different terms? (3) what (in)formal opportunities were there for prisoner actors to be involved? and (4) to what extent were prisoner actors involved in reporting on the incidents and making recommendations? We now consider the three translations.

## Findings

### The prisoner translation

The first, bottom-up translation, involved Strangeways prisoners, the prison and the media. Prisoners sponsored the project, problematising prison conditions through spoken discontent and defined the initial means of resolution by brandishing weapons. Prisoner voice was facilitated by the 309 prisoners in the chapel, which offered an opportunity to define a problem to an interested audience. This large audience of peers who were willing to listen and the prisoners’ access to weapons and masked disguises amplified the power of the problematisation:

An inmate came down the centre aisle [of a chapel service] and took the microphone . . . he began to address the congregation, talking about the hardness of the prison system. Another inmate shouted, ‘you’ve heard enough, let’s do it, get the bastards’ and brandished two sticks . . . other prisoners also brandished weapons and put on masks. (Woolf and Tumim, 1991: 60)

Interessement involved inviting further prisoners to join the conflict and subsequent destruction of property and buildings (Allison, 2010; Woolf and Tumim, 1991). Prisoners who joined the protagonists at this point may have formed a ‘second order body politic’ (Protevi, 2009), where people do not necessarily have control over themselves in a transforming environment with widespread unusual behaviour. In such moments, people can get swept along with the momentum, not necessarily comprehending how or why until much later. Platform, audience, resources and disguise were therefore important for defining the problem and seeking to enrol allies.

Prisoners who enrolled, accepting roles and interests defined by the sponsor, increased the scale of the disturbance: ‘some attacked officers with missiles and sticks, one smashed a bookcase, another threw a fire extinguisher, some grabbed officers’ keys’ (Woolf and Tumim, 1991: 60). In addition to these destructive roles, prisoners also facilitated staff being taken to safety and Woolf and Tumim (1991: 61) names two officers who owe their safety to prisoners who rescued them. As staff sought to retake control over the first 2 days, many prisoners surrendered but a large proportion remained sympathetic to the rioters given the poor conditions they were housed in and a lack of effective methods for complaining (Woolf and Tumim, 1991: 104–105). Enrolment was influenced by the need to challenge conditions and by the presence of an ‘oppositional community’ with a shared ‘critique of the existing order’ and motivation to demonstrate their critique (Ferguson, 1996: 121). Some prisoners became spokespersons, mobilising and translating collective protestor’s interests, and these spokespersons continued to represent the prisoner body after the majority had surrendered (Lord, 2015).

Prisoners’ initial verbal communications were drowned out, as former prisoner Lord (2015) noted,

I was shouting as loud as I could to get our message across [. . . the police] were playing a Barry Manilow song over and over again to drown the shouting . . . As soon as I got the chalkboard and started communicating with the press, the music stopped. (p. 94)

Inscriptions produced by prisoners included written messages on bedsheets and blackboards, which the media photographed and in turn stabilised (e.g. *The Guardian*, 2010). While Woolf and Tumim (1991) says little about these written artefacts, Lord (2015) outlines,

I became the negotiator and tried to get my voice heard, writing messages on the blackboard for all to see . . . protesting the way prisoners were treated in Strangeways. (p. 97)

One such message was ‘Europe treats prison with respect! Why can’t the British bureaucracy do the same, that’s all!’ (Bardsley, 2015: image 1). Core messages transmitted by prisoners were tarnished by initial Ministerial responses:

I utterly condemn the behaviour of the small minority of the prisoners who joined in that orgy of destruction. As the House will be well aware, 183 persons have been convicted or are now awaiting trial on charges including murder and riot. (Kenneth Baker, Home Secretary, 25 February 1991)

However, the ‘textual memory’ provided by prisoners’ artefacts (Tomczak, 2016: 63) remains available in press archives and prisoner writings that powerfully signify discontent (e.g. Lord, 2015).

Mapping this translation reveals how riot enabled prisoners to shape (i.e. initiate) formal external regulation in the absence of other complaint mechanisms. However, riot was limited as a means of mobilising prisoner concerns beyond the prison network. The subsequent official inquiry mobilised prisoner concerns more broadly.

### The Woolf translation

The problematisation for Woolf’s inquiry came from then Home Secretary David Waddington, who set out its terms of reference:

To enquire into the events leading up to the serious disturbances in Her Majesty’s Prison Manchester which began on 1 April 1990 and the action taken to bring it to a conclusion. (cited by PRT, 1991: 1)

Woolf wrote to staff and prisoners seeking evidence on 1 May 1990 and replies were analysed by the inquiry team (PRT, 1991: 2). Few prisoners submitted to Woolf’s interessement, with 16% of Strangeways prisoners responding. The report framed this as ‘a striking and remarkable result’, given that ‘direct mail’ specialists are delighted with a 1%–5% response rate, that there were difficulties finding dispersed prisoners, and a relatively short time to respond (Woolf and Tumim, 1991: 473). However, the spokesperson (Woolf) did not acknowledge that inviting letters to be posted through the institution which imprisons people is problematic, nor appreciate that reading and writing difficulties affect up to 70% of prisoners (Jones and Manger, 2019). There was also no opportunity for prisoners to mobilise or ‘sense test’ Woolf’s analysis and findings, only to submit their written words for interpretation by the inquiry team and to be represented through the report. The Woolf report is almost 600 pages long but included zero prisoner letters in their original form.

The inquiry team created explanatory themes and suggested solutions from the submissions. The prisoner and officer letters contained some remarkable ‘hidden harmony’ (Mazur and Sztuka, 2021). Both groups highlighted overcrowding, poor conditions and sanitation as stimuli for the riots. In 1990, Strangeways was certified to hold just 970 prisoners but held 1647. Both groups also proposed smaller prisons, improved sanitation and better complaint procedures as solutions. Specifically, prisoner letters offered the following explanations for the riot: *poor sanitation and* ‘slopping out’, *overcrowding, being locked in cells for most of the day, poor food* and *poor staff attitudes* (Woolf and Tumim, 1991: 474–475). Regarding conditions:

The toilets and washrooms were degrading and filthy. [. . .] The water hot and cold that you got in buckets and jugs was used to wash your body, items of clothing and cleaning your cell. These same jugs, buckets then had to contain water for drinking and washing knives, forks, spoons. (An extract from Woolf and Tumim, 1991: 474)

Prisoners suggested resolutions including *improved conditions*, (*collective*) *grievance outlets, separate therapeutic centres for vulnerable prisoners, more incentives and responsibilities, smaller prisons*, and *more bail hostels* (Woolf and Tumim, 1991: 476–477).

Prison officers highlighted problems including *staff shortages, poor prison conditions; media coverage; transfers of prisoners; lack of discipline; lack of leadership; lack of precautions; appeasement of inmates; prisoner mix; roof access; local prisons being too big; poor staff-inmate relationships* and *poor rehabilitation* (Woolf and Tumim, 1991: 502). Suggested solutions included *enough staff on duty; improved conditions; prompt use of force; better equipment and (restraint) training; key/roof security; discipline; segregating troublemakers; censoring media; separating vulnerable prisoners*; and *better grievance procedures* (Woolf and Tumim, 1991: 511).

Woolf carried the points of agreement between prisoners and staff regarding conditions, crowding and grievances into his 12 main recommendations, proposing, (7) no prison should exceed certified level accommodation; (8) sanitation for all inmates, (10) dividing prisoners into smaller units; (12) improving justice in prisons and an independent complaints adjudicator. Prisoners’ and officers’ voices therefore amplified one another, shaping Woolf’s ‘interessement’ and the enrolment of some shared concerns. However, there were also points of (partial) agreement not clearly addressed by the inquiry. For example, both parties suggested separating ‘vulnerable’ prisoners from the main population, with prisoners suggesting they be placed in therapeutic settings. Prisoners and officers also both referred to relationship problems, with prisoners critiquing staff attitudes and officers citing a lack of discipline, too much appeasement and poor relationships. While Woolf addressed material issues such as infrastructure and administration, the location and treatment of ‘vulnerable’ prisoners and the contradictory perspectives about prisoner-staff relationships were not translated into recommendations. This avoidance of contradiction was a missed opportunity (Arendt, 1958; Follett, 1924). In collaborative, participatory approaches difference is at ‘the heart of the relationship’ and parties must confront differences in order to re-evaluate values, enabling mutual evolution and unforeseen solutions (Shapiro, 2003: 589–590). Arendt similarly argued that commitment to achieve change is found through disagreements (Bay, 2012: 5). Recognising the plurality in prisoner and officer views may have facilitated exploration of solutions to some of the more complex issues including racism.

Woolf included a single page on ‘race relations’, noting that ‘complaints of racial discrimination from prisons are made regularly to the Commission of Racial Equality’ and considerable disquiet was expressed *by prisoners* about race relations (Woolf and Tumim, 1991: para. 12.131). Despite this statement, little else is written about race and the inquiry’s 12 prominent recommendations made no mention of the issue. Although 1991 preceded reports including the MoJ Race Review (2008) and Lammy (2017), in 1981, Lord Scarman’s inquiry into the Brixton riots highlighted the importance of tackling racial discrimination. In 1989, Genders and Player interviewed randomly selected prison officers and found ‘a mere six officers out of 101 did not refer to black prisoners in racist or pejorative terms’ (Sim, 1994: 38). By 1999, Sir William Macpherson concluded the official inquiry into the death of black teenager Stephen Lawrence, finding that Metropolitan Police incompetence could only be explained by ‘pernicious and persistent institutional racism’ (Hall, 1999: 187). Despite evidence of widespread racism in Britain’s prisons and police force throughout the 1980s, and regular complaints from prisoners, Woolf diverts attention from this substantive issue. Had (Woolf and Tumim 1991: 474) examined prisoner references to ‘poor staff attitudes’ (Woolf and Tumim, 1991: 474) in more detail and included the voices of black (former) prisoners, his recommendations could have engaged with and challenged the discrimination which continues to shape prisons and the criminal justice system today (Lammy, 2017).

Woolf provided an opportunity for prisoner participation and achieved a 10%–20% response, or enrolment rate, across rioting prisons (Woolf and Tumim, 1991). However, participation was limited by the literacy requirement, the team’s representation of prisoners’ concerns and the lack of opportunities for prisoners to ‘sense check’ analysis. Complex points of disagreement between staff and prisoners and entrenched forms of discrimination went unaddressed within this translation, which started with some participation but became increasingly hierarchical and professionalised. We now consider the third and final translation.

### Voluntary sector translation

The PRT report’s problematisation included the riot, the prison conditions that prompted it and the piecemeal implementation of Woolf’s recommendations since 1991. While prisoner participation was limited in this translation, the issues that prisoners raised in the Woolf inquiry informed the evaluation. For example, PRT’s review of Woolf’s fifth recommendation revisits prisoners’ evidence that there was a lack of justice and fairness in their treatment (Day et al., 2015: 14). Woolf recommended a ‘contract’ of expectations between prison and prisoner, ensuring greater consistency. This proposal evolved into the problematic Incentives and Earned Privileges (IEP) scheme, which links prisoners’ behaviour to privileges. However, the scheme has been subject to several reviews, including Justice Secretary Grayling’s ban of books for prisoners (Day et al., 2015; MoJ, 2013). PRT’s review highlighted two means of regulation: judicial review, which enabled the book ban to be overturned and guidance available through PRT’s advice and information service. Prisoners and families reported concerns about the IEP scheme through this telephone service, which led to the charity’s report ‘Punishment without purpose’ and inscription of critique (PRT, 2014). The House of Commons Justice Committee (2015) subsequently noted that the IEP scheme contributed to deteriorations in prison safety and the Prison Governors’ Association denounced the scheme as ‘morally wrong’. This translation therefore illustrates how prisoner concerns (through judicial review and sharing information with a charity advice service) can create larger networks, stimulating or aligning with critiques from politicians and governors to amplify a message and enrol others in the translation of thought or action. However, the IEP scheme endures and the translation is unfinished, perhaps because PRT as sponsor has not clearly defined others’ roles and a means of resolution, or because there is no clear spokesperson amid multiple actors.

Prisoners were passive in PRT’s translation, being involved only through their past words and actions. For example, the report opens with a description of prisoners taking control of Strangeways and later cites a prisoner commenting on the continuing lack of toilet provision. Prisoner voices in this report were filtered and prioritised by the charity and prisoners were not able to submit to, negotiate or refuse their enrolment and mobilisation by PRT. However, this free 39-page document available on PRT’s website inscribes Woolf’s recommendations, which is not freely accessible in digital or paper format at the time of writing. The PRT report outlines ways that prisoners can seek to regulate prisons, including judicial review and charitable advice services. Through these mediums, prisoners might join larger networks of actors (e.g. charities, inspectors, staff collectives, parliamentary committees) who may amplify their concerns and act as powerful spokespeople. However, it offered no opportunities for prisoners to actively define or negotiate problems or solutions. Prisoner views were not as prominent as those of more powerful actors (e.g. Lords, Ministers and the charity). Moreover, serving prisoners are unlikely to be able to read it due to limited Internet access within prisons.

## Towards participatory regulation

We have argued that the Strangeways prison riot (1990), Woolf and Tumim report (1991) and Strangeways 25 Years On report can be conceptualised as translations which individually and collectively attempted to regulate prisons. By analysing them, we traced uneven levels of prisoner voice and action throughout the four phases from problematisation to mobilisation. Considered as rungs on a ladder of citizen participation (Arnstein, 1969), prisoners’ roles across these translations shifted from rioter to letter writer in Woolf, to only report subject in PRT, hence hence their participation levels decreased over time. ‘Degrees of citizen power’ are at the top of Arnstein’s ladder and include partnership, delegated power and citizen control. The 1990 riots briefly offered prisoners some increased control over their environment and an ability to reach a broader audience and inscribe messages of ill-treatment. This degree of participation was not repeated, as the Woolf inquiry invited a form of consultation through letters and testimony. The PRT review informed on progress against prisoners’ previous contributions through the charity’s eyes. The decreasing degree of prisoner participation in these official documents over time is important to acknowledge. However, critics of Arnstein’s ladder argue that the linear, hierarchical model fails to capture the dynamic nature of user involvement (Tritter and McCallum, 2006). An alternative ‘mosaic’ analogy, represents successful user involvement as a system connecting diverse individuals and groups at local, organisational, and national levels. This reveals potential for sharing experience, knowledge and harnessing multiple perspectives (Tritter and McCallum, 2006). We now consider some possibilities for connected, participatory prison regulation, drawing on the strengths of the translations we have examined and avoiding their weaknesses.

The Woolf inquiry happened 30 years ago. Human rights now provide a dominant framework for regulating prisons (Armstrong, 2018) and contemporary prison governance includes some opportunities for prisoners to contribute to decision-making forums and deliver services directly (Buck et al., 2021). Yet, the PRT review provides a reminder that many prisons are still overcrowded and under-resourced and prisoner concerns are not necessarily routinely included in well-intentioned regulation processes. In response, we draw on the strengths and gaps mapped above to pose questions for prison regulators about how their activities include and exclude prisoner perspectives. We include prisoners and former prisoners because different insights will come from people currently imprisoned and those who have had time to reflect at a distance from the oppression of prison. Our questions are informed by the four phases of translation and could provide an audit tool to guide different regulators, including prison inspectors and monitoring boards, ombudsmen, public inquiry teams, voluntary sector organisations and media reporters.

1. *Problem definition* (problematisation) – How are (former) prisoners informed that they can (seek to) regulate prisons, including the variety of platforms available, their opportunities and distinct risks? How are (former) prisoners involved in identifying problems to be solved? How are diverse prisoner perspectives included, for example, black and ethnic minority prisoners, LGBTQ+ prisoners, foreign national prisoners and (learning) disabled or mentally ill prisoners? Do participation methods offer an inclusive ‘continuum of opportunity’ (Weaver, 2019), including spoken and written forms, anonymous routes and forms of organisation based on democratic power?
2. *Building partnerships* (interessement) – How are (former) prisoners (and staff) supported to build and maintain problem-resolution partnerships inside and outside of prison? Are there spaces for *dialogue* between diverse stakeholders? How are the constraints of monologue avoided? Is there focus on synthesised recommendations, which acknowledge agreement *and* disagreement? Do regulators connect with other regulatory actors to amplify shared concerns across organisations and sectors (Tomczak, 2021)?
3. *Clear roles* (enrolment) – Are (former) prisoners *active* in formal regulation networks? Are there opportunities to identify problems, (co)design solutions, and evaluate progress with plans? Are peer support networks available? How does involvement avoid tokenism and placation and promote partnership working and citizen control? Are there pathways to develop (former) prisoners as leaders?
4. *Sharing learning* (mobilisation, spokesperson) – Are regulators’ findings and recommendations accessible to people in prison, including those who cannot read or speak English or who have reduced mental capacity? Are (former) prisoners involved in the dissemination of learning (in written/visual/sound/other creative forms)? Do prisoners assess and report on (lack of) progress with recommendations?

Through these questions, we aim to encourage multisectoral regulators to harness the often-overlooked contribution that (former) prisoners can make, individually and collectively, to analyse problems related to prison and decisions about solutions.

## Conclusion

The 1990 Strangeways riot saw prisoners actively regulate prisons, with significant institutional consequences but dire personal and social costs. One prisoner died and 147 prison officers and 47 inmates were injured. Many more people were terrified, including prisoners and families waiting for news outside. While significant harm was caused by and to prisoners involved in the riot, we argue that these events produced valuable learning and material for reflection, which could be used to underpin safer forums for prisoners to contribute more effectively to prison regulation.

Prisoner-stimulated regulation through riot in 1990 communicated concerns about ill-treatment. Speech offered an accessible medium, yet written forms were adopted when their speech was silenced. Prisoners obtained platforms, audiences, instruments of power and anonymity, which amplified their messages. However, all these features can and should be facilitated outside of riot. Prison councils and democratic therapeutic communities, for example, provide platforms for prisoners to meet in groups, gain democratic power, and contribute to prison operations (Bennett and Shuker, 2018; Weaver, 2019). The Woolf inquiry provided an opportunity for prisoners to communicate directly in writing and investigators included some of these suggestions in their recommendations. This direct correspondence approach could fruitfully be adopted by other regulators, who could also enrol prisoners in mobilising messages (e.g. communicating messages in spoken, written, and creative forms). The PRT translation identified judicial review and charitable advice services as routes for prisoners to participate in regulation. It also highlighted how networks of actors (e.g. charities, inspectors, justice committees) can valuably amplify concerns. The PRT report was free and relatively accessible (to those with Internet access and good literacy). By outlining regulation opportunities and being publicly available, this translation facilitated network building between interested parties.

Significant regulatory frameworks could be harnessed for more substantive penal reforms. Following Tomczak’s (2021) call for multisectoral actors to form denser vertical and horizontal networks to work together and advance issue-based prison regulation, we argue that prison regulation could be more effective and efficient at all levels if the strengths within each of these networks (i.e. prisoners, formal regulators and the voluntary sector) were recognised and harnessed. Including prisoner voices throughout regulatory processes acknowledges that bureaucratic regulators alone may not be able to provide effective or legitimate regulation (Haber and Heims, 2020). Participatory forms of prison regulation could respond to the negative social justice implications of excluding people from matters directly affecting their lives (Watson and Fox, 2018). Prison regulators could benefit from more engagement with people with lived experiences of prison *throughout* translations: from defining problems to being spokespersons for collective solutions, as the community experiencing the phenomena is the site where ‘local knowledge’ is discovered (Fals Borda, 1988) and viable solutions provided (Peralta, 2017). Future regulatory activities could create clear roles for (former) prisoners to work as partners in problem identification and solutions, highlight ways to get involved (using digestible formats), broker broader connections for positive social change, and consider ways to reach serving prisoners through inside news forums. To encourage such practices, we invite regulators to utilise our four-step self-audit as a starting point on the path towards participatory regulation.

## Notes

1.
https://www.theguardian.com/society/2014/dec/05/prison-book-ban-unlawful-court-chris-grayling
